# Optical Flow and Driver's Kinematics Analysis for State of Alert Sensing

**DOI:** 10.3390/s130404225

**Published:** 2013-03-28

**Authors:** Javier Jiménez-Pinto, Miguel Torres-Torriti

**Affiliations:** Department of Electrical Engineering, Pontificia Universidad Católica de Chile, Vicuña Mackenna 4860, Casilla 306-22, Santiago, Chile; E-Mail: jejimenp@puc.cl

**Keywords:** fatigue detection, alert state sensing, driver assistance, head tracking, PERCLOS, driver's kinematics

## Abstract

Road accident statistics from different countries show that a significant number of accidents occur due to driver's fatigue and lack of awareness to traffic conditions. In particular, about 60% of the accidents in which long haul truck and bus drivers are involved are attributed to drowsiness and fatigue. It is thus fundamental to improve non-invasive systems for sensing a driver's state of alert. One of the main challenges to correctly resolve the state of alert is measuring the percentage of eyelid closure over time (PERCLOS), despite the driver's head and body movements. In this paper, we propose a technique that involves optical flow and driver's kinematics analysis to improve the robustness of the driver's alert state measurement under pose changes using a single camera with near-infrared illumination. The proposed approach infers and keeps track of the driver's pose in 3D space in order to ensure that eyes can be located correctly, even after periods of partial occlusion, for example, when the driver stares away from the camera. Our experiments show the effectiveness of the approach with a correct eyes detection rate of 99.41%, on average. The results obtained with the proposed approach in an experiment involving fifteen persons under different levels of sleep deprivation also confirm the discriminability of the fatigue levels. In addition to the measurement of fatigue and drowsiness, the pose tracking capability of the proposed approach has potential applications in distraction assessment and alerting of machine operators.

## 1. Introduction

Road traffic accidents are one of the main non-health related causes of death. The data and statistics of the World Health Organization [1] show that about 2.8% of non-health related deaths are due to suicide, violence and wars, while 2.1% are attributed to traffic accidents, even surpassing nutritional deficiencies, which account for about 0.9% of world deaths [2]. On the other hand, the social and economic cost of traffic incidents has been estimated to be 1% of the gross national product in low-income countries, 1.5% in middle-income countries and 2% in high-income countries, totaling a global cost of US$518 billion per year [3]. Unlike many diseases and health problems for which there is no cure, traffic accidents can be reduced if proper education, law enforcement and engineering practices are implemented [4,5].

Several studies exist that analyze physiological cues associated with a driver's awareness and state of alert [6–8]. Measuring some of the cues, especially physiological ones, such as EEG, ECG, EOG, blood pressure and body temperature [9,10], may require invasive techniques, and despite some recent improvements in the development of highly sensitive and less intrusive electrodes for ECG monitoring [11], their use as a reliable metric is difficult, because signals like ECG often exhibit significant inter-individual variabilities that depend on factors, such as age, gender, spatial ability and intro-extroversion [8]. Other methods monitor the driver's steering performance (reaction rates and unexpected lane departures) to warn the driver. However, despite claims that these approaches have low false alarm rates, it is also known that these methods fail to predict micro-sleeps, and there is not enough evidence to support these methods as a reliable way of measuring the driver's state of alert [6,12,13]. Fortunately, there are many behavioral changes that provide reliable visual cues of the driver's state of awareness that can be measured in a non-invasive manner with image processing techniques, namely, eye-blinking frequency and percentage of eyelid closure over time (PERCLOS, [14,15]), yawn frequency, head movement and eye-gaze, among other facial expressions. The vision-based approaches must rely on specific techniques to detect the driver's head, face and eyes. Some methods employ intensity and color analysis techniques to segment the parts of the head from the image [13,16–21], while several other approaches rely on the Viola-Jones detector [22–28]. Some approaches only track the eyes, while others focus on particular facial cues, such as yawning [19,29]. A limitation of the approaches based on color analysis is their sensitivity to illumination conditions and the fact that they often cannot be applied at night [30,31]. This has motivated some researchers [30–34] to use near-infrared (IR) cameras, exploiting the retinas' high reflectivity to 850 nm wavelength illumination [35,36]. On the other hand, the performance of the approach is also determined by the type of classifier used to process the features extracted from the image. For example, some approaches employ neural-networks to classify segmented regions as the head and its parts [37,38], while others rely on a variety of template matching schemes [29,39–42]. For a recent survey on drowsiness detection systems, the reader is referred to [43].

This work presents a non-invasive sensing approach for driver fatigue and attention measurement, which is based on a standard charge-coupled device (CCD) camera with an 850 nm near-infrared (IR) filter and a circular array of IR LEDs. The proposed approach draws on ideas by the authors presented in [44], which introduces the use of face salient points to track the driver's head, instead of attempting to directly find the eyes using object recognition methods or the analysis of image intensities around the eyes, as the majority of the exiting approaches to fatigue assessment. An advantage of salient points tracking, as proposed in [44], is that the approach is more robust to occlusions of the eyes whenever they occur, due to the driver's head or body motion. On the other hand, the grid of salient points can be tracked with a low processing cost using the Lukas-Kanade algorithm for sparse optical flow computation. The measurement of the salient points' optical flow provides valuable information for computing changes in the driver's gestures, e.g., eyebrow raisings and yawning. However, it is to be noted that prior results have shown that eyebrow raisings and yawning do not have a sufficiently good correlation with fatigue and thus cannot be used as the main predictor of fatigue. Different works have studied the connection between yawning and vigilance showing that: (i) yawning indeed occurs during progressive drowsiness, which is compatible with the notion that yawning is triggered by states of low vigilance; however, yawns were not triggered nor followed by a specific autonomic activation [45,46]; and (ii) the correlation between facial muscle activity and drowsiness is lower (60–80%) than that of blinking (>80%) [47]. Moreover, it is possible to exhibit high levels of yawning without necessarily being in a hypovigilance state [48]. Therefore, facial muscle activity (including yawning and eyebrow raisings) offers little predictive information pertaining to sleep onset [14]. In fact, sleep can occur without yawning or even before any significant change in muscle activity or tonicity [14]. It has been shown in [49] that also head movement distance and velocity have a stronger correlation (>80%) to sleepiness than the correlations in [47] for changes in facial expression (60–80%). Because of these reasons, and the fact that the percentage of time that the eyes are closed (the eyelids cover the pupils at least 80% or more) over a given period of time (PERCLOS [14]) has a significantly stronger correlation to fatigue [15], efforts should be placed on improving head and eye tracking methods. Furthermore, recent works [50,51] confirm that among the different ocular variables, PERCLOS is the most effective to prevent errors or accidents caused by low vigilance states, thus confirming the original observations and findings reported in [14,15]. In this context, the contributions and novelty of this paper can be summarized as follows. A kinematic model of the driver's motion is introduced to obtain the pose of the driver described by five degrees of freedom (lateral tilt, nod and yaw of the head about the neck and frontal and lateral tilt of the torso). The use of the driver's kinematic model allows one to reach an outstanding performance, with an almost 100% tracking rate of the eyes. A high tracking rate is key to the computation of the PERCLOS, since computing the PERCLOS requires the knowledge of where the eyes are and whether they are open or closed. Another contribution of this work is the use of the driver's observed interpupillary distance (IPD) to estimate the distance from the driver's head to the camera (up to a scale factor), thus the approach yields the driver's motion in 3D space. It is shown that tracking in 3D space the back-projected salient points (from 2D image space to 3D space) is equivalent to tracking points on the 2D image space when the knowledge of the distance between the driver and the camera is available. Therefore, an equivalent result to that of tracking the salient points in 3D space is possible by tracking points in 2D space together with the computed driver-camera distance when the salient points are assumed to be a set of coplanar points lying on the facial tangent plane. Although the salient points do not belong to a coplanar plane; their difference in driver-camera distances are negligible, and therefore, the incidence of this assumption in the performance of the system is negligible for practical purposes. Furthermore, the driver's kinematic motion model allows one to implement an extended Kalman filter that simplifies the tracking of the points in the image space (only the five pose angles need to be estimated with the filter, instead of applying a filter to each of the salient points in the image). Finally, the experiments involving a group of 15 persons—five of which were deprived from sleep for more than 24 h before the driving tests in the simulator; another five were asked to sleep only four hours the night before the test, and the remaining five had a full eight hours rest—demonstrate that the PERCLOS measure is strongly related to fatigue and somnolence and, thus, can be reliably used to warn drowsy and distracted drivers about their impairment and diminished ability to drive correctly.

In contrast to [22,31,32], the proposed approach only employs the Viola-Jones detector to find the driver's face at the initialization stage and only every certain number of frames whenever some reset conditions are satisfied (see Section 2.2.4). Moreover, the proposed approach relies on the tracking of a set of salient points, the driver's kinematic model and the measurement of the IPD, which combined allow for higher eye tracking rates despite occlusions and fast changes in pose. The idea of simultaneous shape modeling and tracking has also been suggested in [52], which uses also an incrementally built texture model, so that the tracker may operate without active illumination. However, the latter also does not include the driver kinematic model, nor uses the Lukas-Kanade to build and track a grid model like the one employed here, but rather builds an appearance and shape model, which allows for small track losses. Finally, it is to be noted that with the proposed approach in this work, it is possible to recover the driver's pose and 3D information using a single camera and the measurement of the IPD. To the best of our knowledge, this is an important aspect not considered yet in the existing published research.

A detailed description of the proposed approach is presented in the next section, followed by an explanation of the experimental methodology in Section 3. The results presented in Section 4 show that our approach yields high eye-tracking rates and reliable confirmation of the driver's state of alert, as inferred from the measured PERCLOS. The main conclusions are presented in the last section.

## 2. State of Alert Sensing

The proposed system design for sensing a driver's state of alert can be divided into three stages: (i) image capture; (ii) driver detection and pose tracking; and (iii) driver vigilance measurement. The image acquisition stage employs a standard low-cost charged-coupled device (CCD) surveillance camera with a near-infrared 850 nm filter and a circular array of infrared light emitting diodes (LEDs). The driver detection and tracking module employs a regular computer to process the images and obtain the driver's motion and facial cues, such as blinking, yawning and head pose. Finally, the driver vigilance measurement stage is responsible for computing the driver's drowsiness and attention levels using information about the percentage of time over a given period that the eyelids cover 80% or more of the pupils (PERCLOS [14,15]) and the head pose obtained in the previous stage. Other cues, such as blinking frequency and yawning, can also be computed; however, they are not taken into account for triggering alarms in the current system, because they were found to have weak correlations with vigilance and drowsiness levels in previous work by the authors [44].

### 2.1. Image Capture and Preliminary Processing

The camera employed to acquire the driver's images has a 640 × 480 CCD array, a lens with focal length f = 4 mm and a circular arrangement of 26 infrared LEDs. The camera was modified to include an 850 nm band-pass filter lens that covers both the image sensor and the IR LEDs. The purpose of the near-IR filter lens is two-fold. The filter lens serves to improve the rejection of external sources of infrared radiation and reduce changes in illumination produced by the sun as the car moves. On the other hand, using the filter facilitates the detection of the pupils, because the retina is highly reflective of the near-IR illumination of the LEDs, resulting in a “red-eye” effect similar to that of standard photography. A video of the images from the camera is generated using a composite-video to USB converter. The converter allows one to capture the frames directly on a PC at a rate of 30 fps.

**Figure 1.**
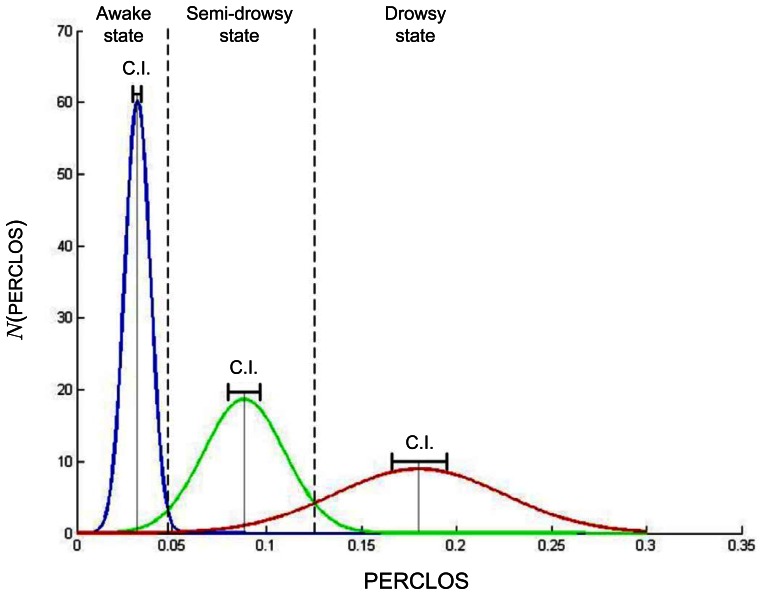
Normal distributions of the different states of alert calculated for the awake, semi-drowsy and drowsy subjects, showing the means, the confidence intervals (CI) and the identification threshold between one level of alertness and the other.

The main tasks of the preliminary processing algorithms are the reduction of the image size to 384 × 288 and the execution of the Viola-Jones face detection algorithm when the system initializes to provide for a gross estimate of the head location. Reducing the images allows the system to achieve a frame rate of 16.5 fps using a 2.2 GHz CPU, which is a processing rate sufficient for on-line real-time operation of the system. It is to be noted that for fatigue detection purposes, any vision-based system should have a sampling and processing rate of at least 5–10-times per second, because:(i) eye blinks last between 0.1–0.4 seconds [53]; and (ii) microsleeps last from a fraction of a second up to 30 seconds, but more typically three to 14 seconds, according to the experimental studies in [54]. Furthermore, the resolution of the PERCLOS measurements computed at 16.5 fps is enough to obtain clearly distinguishable states of fatigue, as shown by our results (see, for example, Figure 1).

### 2.2. Driver Detection and Pose Tracking

**Figure 2.**
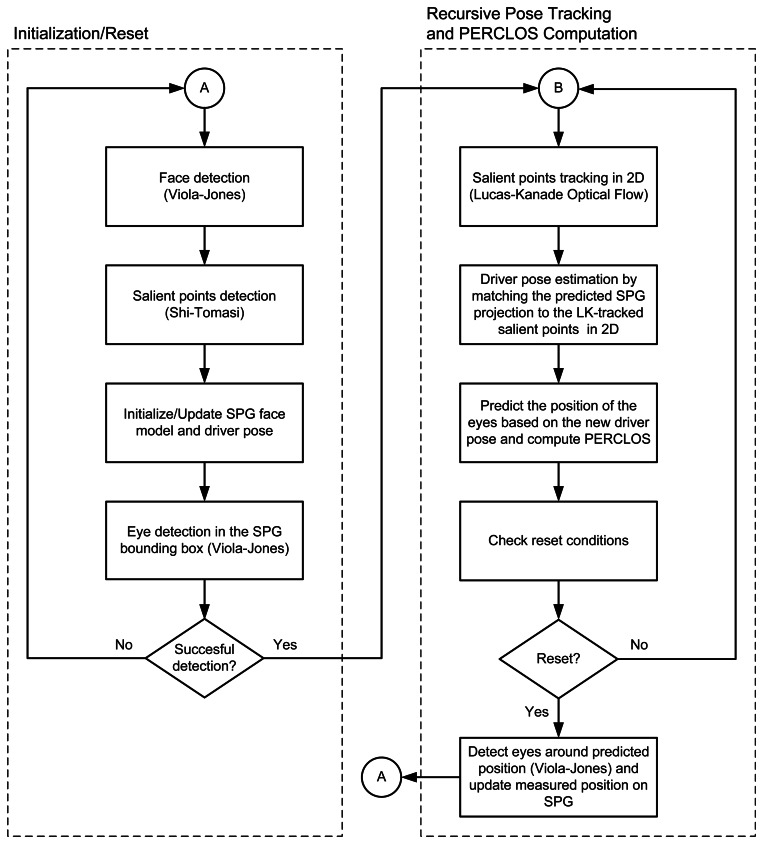
Flow chart of the proposed method.

Correct driver detection and tracking is key to the measurement of driver attention and fatigue. The driver detection and tracking process can be divided into an initialization phase, in which the driver's nominal pose is computed, and an iterative pose estimation and tracking phase, as shown in Figure 2.

The initialization step starts with the detection of the driver's face using the well-known Viola-Jones object recognition method [55]. A bounding box for the head *ℬ^h^* is computed from the face position information. Distinctive corner points within the face that are good points for tracking regardless of their position, including points that change from face to face, such as eyebrow corners, freckles, moles, scars, beard, *etc.*, are found using the Shi-Tomasi detector [56]. A grid that will be referred to as the *salient points grid* (SPG) is next computed using the distinctive points. The SPG is modeled as a group of coplanar points in 3D space that make up a non-deformable mesh structure having five degrees of freedom (DOF), which replicate the driver's head-torso kinematics. The initialization process ends with the detection of the eyes using the Viola-Jones algorithm trained for such purpose. The search is carried out in an area defined within *ℬ^h^*, where the eyes are expected to be found, on average, according to the head's anatomy.

Once the initial location of the SPG in 3D space is inferred using the driver's observed interpupillary distance (IPD), the driver's pose (position and orientation) is solved by computing an SPG motion, such that the projection of the SPG points from 3D space onto the 2D image plane coincides with the salient points tracked in the image using the Lukas-Kanade method [57]. The driver's kinematic model, the SPG and the driver's pose estimation method will be explained in greater detail in Sections 2.2.1–2.2.3, respectively. Finally, on each iteration, the driver's eyes are sought within expected regions, according to the newly determined head pose. Tracking the driver's pose significantly improves the ability to locate the driver's eyes, even if for several consecutive frames the eyes cannot be directly detected from the image analysis. Continuous tracking of the head, and, thus, the eyes, greatly simplifies locating the pupils with accuracy and determining whether they are visible or not due to blinking or head rotations, particularly sideways. The analysis of the PERCLOS [14,15] to provide an indication of the driver's state of vigilance will be explained in more detail in Section 2.4.

#### 2.2.1. Driver's Kinematic Model

**Figure 3.**
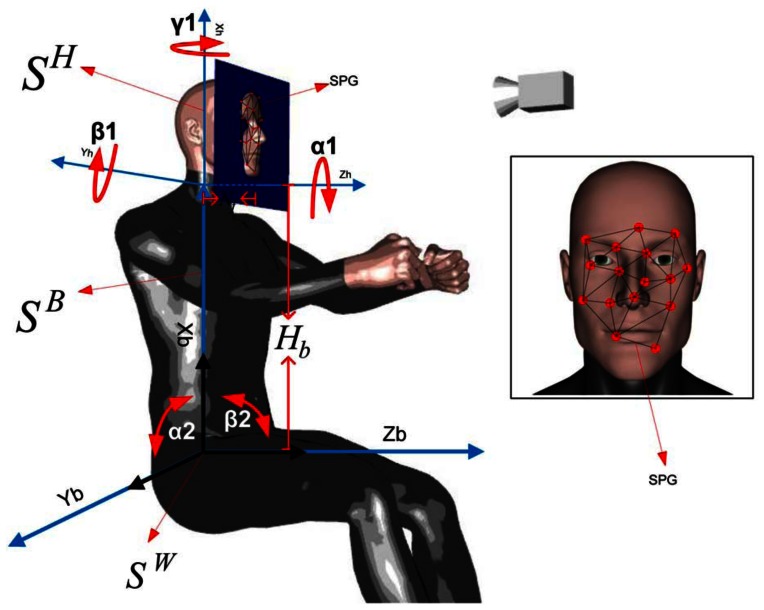
Driver's kinematics and the salient points grid (SPG) fixed to the driver's face.

The motion of the driver can be decomposed into head motion and torso motion, as depicted in Figure 3. The head motion is achieved by a complex system of muscles, tendons and ligaments in the neck that support and connect the cervical spine with the skull. The different forward/lateral extensor, flexor and rotator muscles allow the head to rotate sideways, bend forward, backward or lean sideways. These movements can be characterized by a 3-DOF spherical joint. On the other hand, the torso motion can be described by a two-DOF universal joint located at the waist that allows the driver to bend forward or backward (frontal tilt) or lean sideways (lateral tilt). For practical purposes, subtle pose changes that are possible with the complex musculoskeletal system of the back are assumed to have been taken into account as part of the waist frontal/lateral tilt or the neck motion.

The driver's upper body kinematics decomposed into torso and neck movements allows one to describe the motion of the head with reasonable fidelity for adequate tracking purposes. Thus the proposed motion model employs three coordinate frames: *S^H^*, *S^B^*, *S^W^* for the head, body and world (fixed base) coordinates, respectively (see Figure 3). Obtaining the location of the head relative to the fixed world coordinate point is done by translation and rotation transformations between the coordinate frames.

For purposes of deriving the coordinate transformations between coordinate frames, first consider that *S^H^*, *S^B^* and *S^W^* are spatially coincident, *i.e.*, are aligned and share the same origin point. The first step to express the position of points on the driver's face relative to *S^W^* consists in computing a transformation to express face points with coordinates in the *S^H^* as points relative to the body frame, *S^B^*. This transformation takes into account the neck motion and yields the head pose relative to the torso by rotating *S^H^* (and the points fixed to *S^H^*) about the z-axis, y-axis and x-axis of *S^B^* by angles, *α_1_, β*_1_ and *γ*_1_, respectively. The rotated *S^H^* must be then translated by a distance *H_b_* along the y-axis of *S^B^* These transformations yield the following homogeneous transformation matrix:

(1) $$p_{h}=\left[\begin{array}{cccc} c(\alpha_{1})c(\beta_{1}) & c(\alpha_{1})s(\beta_{1})s(\gamma_{1})-s(\alpha_{1})c(\gamma_{1}) & c(\alpha_{1})s(\beta_{1})c(\gamma_{1})+s(\alpha_{1})s(\gamma_{1}) & H_{b}\\ s(\alpha_{1})c(\beta_{1}) & s(\alpha_{1})s(\beta_{1})s(\gamma_{1})+c(\alpha_{1})c(\gamma_{1}) & s(\alpha_{1})s(\beta_{1})c(\gamma_{1})-c(\alpha_{1})s(\gamma_{1}) & 0\\ -s(\beta_{1}) & c(\beta_{1})s(\gamma_{1}) & c(\beta_{1})c(\gamma_{1}) & 0\\ 0 & 0 & 0 & 1 \end{array}\right]$$

where the notation *c*(*θ*) = cos(*θ*) and *s*(*θ*) = sin(*θ*) has been used for brevity.

The next step consists in computing the transformation matrix to express the torso motion relative to the base. To this end, *S^B^* is rotated about the *y*-axis and z-axis of *S^W^* by angles α_2_ and *β_2_*, respectively, yielding the body homogeneous transformation matrix:

(2) $$p_{b}=\left[\begin{array}{cccc} c\left(\alpha_{2}\right)c\left(c\beta_{2}\right) & -s\left(\alpha_{2}\right) & c\left(\alpha_{2}\right)s\left(\beta_{2}\right) & 0\\ s\left(\alpha_{2}\right)c\left(\beta_{2}\right) & c\left(\alpha_{2}\right) & s\left(\alpha_{2}\right)s\left(\beta_{2}\right) & 0\\ -s\left(\beta_{2}\right) & 0 & c\left(\beta_{2}\right)c\left(\gamma_{2}\right) & 0\\ 0 & 0 & 0 & 1 \end{array}\right]$$

Finally, for a given driver's pose, described by angles *α_1_, β_1_*, γ_1_, α_2_ and *β*_2_, the location of any point of the SPG *X^H^* ∈ ℝ^4^ with the coordinates referred to *S^H^* can be expressed as a point *X^W^* ∈ ℝ^4^ with the coordinates referred to, *S^W^* using Equations (1) and (2) to calculate:

(3) $$X^{W}=PX^{H},\text{with}\;P=P_{b}P_{h}$$

**Figure 4.**
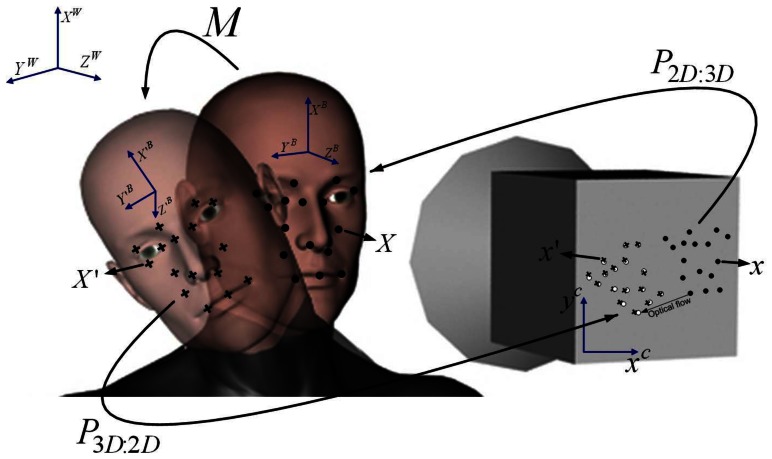
Transformation of the salient points involved on the proposed approach.

It is to be noted that the pose matrix P depends on the pose angles, α_1_, *β*_1_, γ_1_, α_2_ and *β_2_*. For two instants k and *k* +1 corresponding to sampling times *t* and *t* + *T_s_* with sampling period *T_s_* > 0, there will be two associated pose matrices *P_k_* and *P_k_*_+1_, and a transformation *M_k_*_+1_: *P_k_ → P_k_*_+1_ ∈ ℝ^4−4^ that maps points $X_{k}^{W}$ of the SPG at time k onto points $X_{k+1}^{W}$ at time *k* + 1. This transformation is illustrated in Figure 4 as mapping *M*.

#### 2.2.2. The SPG and the Perspective Projection Model

**Figure 5.**
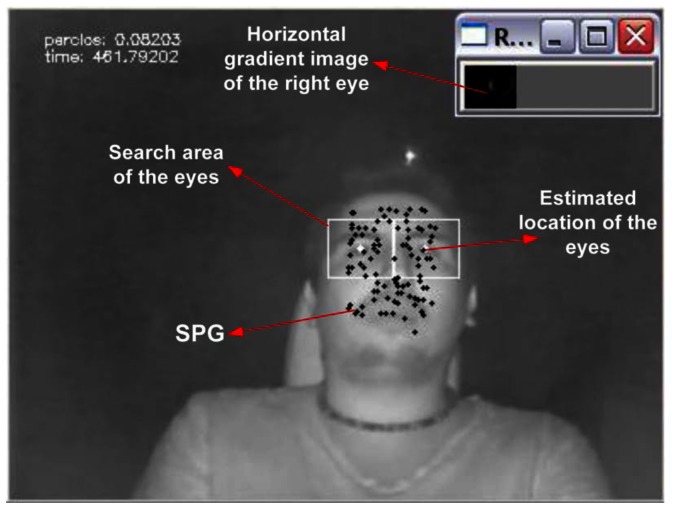
Snapshot of the system running.

Visual tracking in 3D space of any object using a single-camera view is a challenging problem, because depth information is lost due to the perspective projection that maps scene points in 3D space onto image points in the 2D sensor plane of the camera. However, whenever some knowledge of the object's geometry and size is available *a priori*, it is often possible to recover 3D motion and pose information. The proposed driver tracking approach takes advantage of the fact that all salient points of the SPG belong to the driver's head, which for the purpose of the proposed approach, can be regarded as a *rigid object* of *standard size* located at a regular nominal distance from the camera. By *rigid object* it is meant that the skull as a structuring element is non-deformable. Hence, the eyes, the cheek bones and the nose preserve their relative distances with respect to each other. It is to be noted that locally around the eyes and mouth, the face is a deformable (non-rigid) object that changes when the driver talks or makes gestures. However, unlike our prior work [44], here, we are not considering gestures, such as eyebrow raisings or yawning, as the small deviations of SPG points around the mouth can be handled correctly by the Lucas-Kanade tracker. Therefore, for the purpose of the proposed approach, changes in gesture can be neglected, and the SPG can be treated as a set of salient points that can be consistently tracked and that preserve their relative distance in 3D space, as illustrated in Figure 3 and shown for a real driver in Figure 5. By *rigid object* we do not mean the head is motionless or rigidly fixed. On the other hand, while there do exist correlations between the morphological characteristics of bones and the population that tend to follow geographic boundaries often coinciding with climatic zones, the size of the head changes little across different populations and phenotypes for people 18 years or older (see, for example, [58]). The average male head is around 20 × 15 ± 2.2 × 0.7 cm, while the average female head is 18 × 13 ± 1.2 × 1.2 cm. This ±3 cm variation relative to the camera-head operating distance is negligible. Because of this reason, it is possible to consider the head as an object of *standard size*, that changes little from one driver to another, and due to its low variance, its size is very predictable. Furthermore, an important feature of the proposed approach is that it does not require the heads to be exactly equal, because the SPG is created on-line for each person.

Among the salient points, the pupils are two points of special interest, because the IPD provides a reliable reference yardstick of *a priori* well-known and predictable size that is relatively invariant for adult people [59]. For females and males over 18 years of age, the IPD has respectively been estimated to be 62.3 ± 3.6 mm and 64.7 ± 3.7 mm; *cf.* [59]. If *D_p_* is the driver's IPD and *d_p_* is the length of the projected IPD onto the image sensor, using the projective geometry equations, it is possible to find the distance, z_hc_, between the driver's head and the camera as:

(4) $$Z_{hc}=\frac{f}{d_{p}}D_{p}$$

where *f* is the focal length of the camera lens. The accurate knowledge of *Z_hc_* in Equation (4) requires the exact knowledge of the *D_p_*, *f* and the measurement of *d_p_*. However, it is possible to show that even if an error in the knowledge of *D_p_* introduces an error in the estimation of the absolute distance *Z_hc_* between the driver and the camera, the relative displacement error between an initial and a final position is negligible. To this end, consider an erroneous measurement Z̃_hc_, given by:

$${\tilde{Z}}_{hc}=\frac{f}{d_{p}}{\tilde{D}}_{p}$$

due to an erroneous value *D̃_p_* of the IPD. Then, the error *e_Zhc_* between the true distance *Z_hc_* and the erroneous measurement *Z̃_hc_* is given by:

$$\begin{array}{ccc} e_{Z_{hc}} & = & {\tilde{Z}}_{hc}-Z_{hc}\\ & = & \frac{f}{d_{p}}{\tilde{D}}_{p}-\frac{f}{d_{p}}D_{p}\\ & = & \frac{f}{d_{p}}({\tilde{D}}_{p}-D_{p})\\ & = & \frac{f}{d_{p}}e_{D_{p}} \end{array}$$

where *e_Dp_* = *D̃_p_* − *D_p_* is the error in the measurement of driver's IPD. The error *e_Zhc_* represents the *absolute position error* in the measurement of the distance *Z_hc_*. On the other hand, the *relative position error, i.e.*, the error in measuring the displacement of the driver from an initial position *Z_hci_* to a new position *Z_hcf_* due to an error *e_Dp_* in measuring the IPD is given by:

$$\begin{array}{ll} e_{\Delta_{z}} & =\underset{\equiv {\tilde{\Delta }}_{z}}{\underset{︸}{\left(\frac{f}{d_{p_{f}}}{\tilde{D}}_{p}-\frac{f}{d_{pi}}{\tilde{D}}_{p}\right)}}-\underset{\equiv \Delta_{z}}{\underset{︸}{\left(\frac{f}{d_{p_{f}}}D_{p}-\frac{f}{d_{p_{i}}}D_{p}\right)}}\\ & =fe_{D_{p}}\left(\frac{1}{d_{p_{f}}}-\frac{1}{d_{p_{i}}}\right)\\ & =fe_{D_{p}}\left(\frac{Z_{hcf}}{fD_{p}}-\frac{Z_{hci}}{fD_{p}}\right)\\ & =\frac{e_{D_{p}}}{D_{p}}\Delta_{z} \end{array}$$

where Δ*z* = *Z_hcf_* − *Z_hci_* is the driver's displacement along the *z*-axis of the camera, $d_{p_{i}}=\frac{fD_{p}}{Z_{\text{hci}}}$ and $d_{p_{f}}=\frac{fD_{p}}{Z_{\text{hcf}}}$ are the projections of the interpupilar segment *D_p_* when the driver is at an initial distance *Z_hci_* and at a final *Z_hcf_* of the camera, respectively.

From a practical standpoint, an error *e_Dp_* = ±3*σ_IPD_* = ±11.1 mm (three-times the standard deviation of the IPD, *σ_IPD_* ≤ 3.7, [59]), would yield an absolute position error *e_Zhc_* = ±133.2 mm when using a lens of focal distance *f* = 4 mm and a typical camera with a pixel size of ∼ 5 *μm* (*d_p_* ∼ 0.33 mm). On the other hand, for the average driver with an IPD *D_p_* = 63.5 mm, the scaling factor $\frac{e_{D_{p}}}{D_{p}}$ will be about ±0.17. Thus, considering that the driver typically sits about 50–80 cm from the camera, depending mainly on the arms' and legs' length, if the driver moves, for example, from an initial position *Z_hci_* = 65 cm to a position *Z_hcf_* = 55 cm, then Δ*z* = −10 cm and *e*_Δ_*_z_* = ±0.17 10 = ±1.7 cm. Hence, the measured displacement will be in the interval Δ̃*z* &isin (−11.7, −8.7) cm instead of −10 cm in the worst case. In practice, the video acquisition is done at a speed for which driver displacements Δ*z* will be a few millimeters. Therefore, *e*_Δ_*_z_* will be a few tenths of a millimeter between frames, which is negligible for practical purposes, even if in terms of absolute accuracy, and the measurement of the driver position with respect to his or her true position is offset by ±13 cm in a worst case scenario. Moreover, since we are interested in measuring the relative displacement of the driver from the normal driving position, rather than calculating the exact distance between the driver and the camera with absolute accuracy, and considering that the displacement error is negligible, the method can be applied effectively for actual implementation. It is to be noted that if a developer wishes to have an accurate absolute position measurement, then either the IPD must be entered accurately as a parameter of the system or alternative position sensors, such as simple IR proximity sensors or highly accurate PSD-based sensors, should be included. Since the salient points belong to a 3D rigid object, their geometric constraints (e.g., relative distances among the points) are fully satisfied only in 3D space, but not preserved in the image plane, due to the perspective projection and the relative pose change between the driver and the camera. Hence, obtaining the motion of the head and change of driver's pose is possible by finding a transformation matrix *M: X* ∈ ℝ^4^ → *X′* ∈ ℝ that corresponds to the motion of the SPG, such that the mapping of the initial's pose SPG onto the new pose SPG yields a new SPG, whose projection onto new salient points in the image matches the salient points already tracked in the 2D image plane. This idea is illustrated in Figure 4, which shows an initial head pose and a new head pose associated with a motion transformation *M*. The SPG for the initial pose is represented by dots, while the SPG for the new pose is represented by crosses in Figure 4. An inverse perspective matrix *P_2D:3D_*: *x* ∈ ℝ^3^ → *X* ∈ ℝ^4^ allows one to project the salient points in the 2D image back onto the initial head pose. The motion *M* can then be found as the one that produces a new SPG, whose standard perspective projection *P_3D:2D_*: *X′* ∈ ℝ^4^ → *X′* ∈ ℝ^3^ maps the new 3D SPG points correctly onto the new salient points in the 2D image found with the Lucas-Kanade optical flow computation method. The motion *M* is parameterized using the driver's kinematic model Equation (3), while the inverse projective transformation from camera to world coordinates (*P_2D:3D_*) and *vice versa (P_2D:3D_*) are found as explained next.

For clarity of exposition, it is convenient to introduce some notation first. Let *X^W^*, *X^H^* and *X^C^* be any point in the set *S* of salient points that conform the SPG, expressed as homogeneous coordinates in ℝ^4^ referred to as *S^W^*, *S^H^* or *S^C^* (the world, head or camera coordinate frame), respectively. Let *C* ∈ ℝ^4−4^ denote the homogeneous transformation matrix relating the camera position (translation) and orientation (rotation) with respect to *S^W^*, *i.e.*, any point *X^C^* with coordinates relative to the camera frame *S^C^* can be expressed as a point *X^W^* with coordinates relative to the world frame *S^W^* according to:

(5) $$X^{W}=CX^{C}$$

If the camera is located such that its coordinate frame *S^C^* has a *z*-axis that: (i) points towards the driver; (ii) is parallel to the *z*-axis of *S^W^*; and (iii) is contained in the *XZ* plane of *S^W^*, then:

(6) $$C=\left[\begin{array}{cc} R_{S_{x}^{W},\pi }R_{S_{z}^{W},\frac{\pi }{2}} & \begin{array}{c} t_{3\times 1} \end{array}\\ 0_{1\times 3} & \begin{array}{c} 1 \end{array} \end{array}\right]=\left[\begin{array}{cc} \begin{array}{ccc} 1 & 0 & 0\\ 0 & 0 & 1\\ 0 & -1 & 0 \end{array} & \begin{array}{c} h_{c}\\ 0\\ d_{c} \end{array}\\ \begin{array}{ccc} 0 & 0 & 0 \end{array} & 1 \end{array}\right]$$

where *h_c_* is the height of the camera with respect to the seat (plane *Y Z* of *S^W^* in Figure 3), *d_c_* is the distance from the camera's focal point to the *z*-axis of *S^W^* and *R_u,θ_* represents the rotation matrix about axis *u* by an angle *θ*, while *t*_3×1_ is the translation vector from the origin of *S^W^* to the origin of *S^C^*.

Points $X^{C}={\left[X_{x}^{C},X_{y}^{C},X_{z}^{C},1\right]}^{T}$ in homogeneous coordinates of the camera frame *S^C^* can be projected onto the camera's optical plane as points:

(7) $$p={\left[p_{x},p_{y},1\right]}^{T}=\prod X^{C}$$

using the standard pin hole camera model with a perspective projection matrix II given by:

(8) $$\prod =\left[\begin{array}{cccc} \frac{f}{X_{z}^{C}} & 0 & 0 & 0\\ 0 & \frac{f}{X_{z}^{C}} & 0 & 0\\ 0 & 0 & \frac{1}{X_{z}^{C}} & 0 \end{array}\right]$$

where *f* is the focal length of the camera and $X_{z}^{c}$ is the distance between the point *X^C^* and the camera's focal point measured along the *z*-axis of *S^C^*. For simplicity of exposition, here II is the perspective projection matrix for a coordinate frame *S^C^* with origin located at the camera's focal point. If the origin of *S^C^* is displaced from the focal point, then the last column of Equation (8) must also include the translation terms.

The distance *Z_hc_* provides an initial value of $X_{z}^{c}$ for all points in *S*. If the image coordinates (*p_x_*, *p_y_*) of point *p*, corresponding to point *X^C^* and the distance $X_{z}^{c}$ to point *X^C^* are known, then it is possible to define an *inverse perspective mapping* Γ : ℝ^3^ → ℝ^4^ that projects point *p* on the image plane back onto *X^C^* as:

(9) $$\Gamma =\left[\begin{array}{ccc} \frac{X_{z}^{C}}{f} & 0 & 0\\ 0 & \frac{X_{z}^{C}}{f} & 0\\ 0 & 0 & X_{z}^{C}\\ 0 & 0 & 1 \end{array}\right]$$

such that:

(10) $$X^{C}=\Gamma p$$

The back-projection of points *p* onto points *X^C^* of the SPG is illustrated in Figure 3 as a projection *P*_2D:3D_ from 2D to 3D. On the other hand, the standard perspective mapping II projecting 3D SPG points onto 2D points is represented in Figure 3 as the projection *P*_3D:2D_.

#### 2.2.3. Driver Pose Estimation

**Figure 6.**
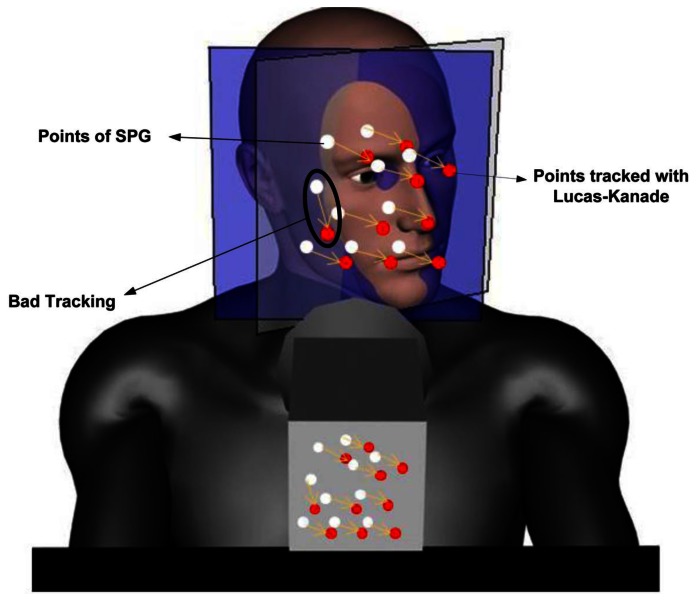
Example of LK tracking between two frames, showing an incorrectly tracked point.

The driver's pose estimation problem consists in finding the pose angles *α*_1_, *β*_1_, γ_1_, α_2_ and *β*_2_ at time instant *k* + 1, given the knowledge of the pose at time *k* and the driver's motion *M_k_*_+1_ at instant *k* + 1 as measured from the image. The proposed approach to estimate and track the driver's pose angles employs the Lucas-Kanade's (LK) method to optical flow computation [57]. The LK method computes a set *S_k_* of salient points, *p_j_*,*_k_*, *j* = 1,2,…,*N*, in an image frame at instant *k* and tracks point-by-point yielding weights *W_j_,_k_*_+1_ and a set *S_k_*_+1_ of salient points *p_j_*,*_k_*_+1_, *j* = 1, 2, …,*N*, in the image frame at instant *k* + 1 corresponding to the points p_j_,*k*, *j* = 1, 2,…,*N*, in the previous frame, as illustrated in Figure 6. The velocity at which corresponding pixels move from *p_j_*,*_k_* to *P_j,k_*_+1_, approximated by *ϕ_j_*,*_k_*_+1_ = (*P_j_,_k_*_+1_ − *p_j,k_*)/*T_s_* for a sampling period *T_s_*, is the so-called *optical flow* of the image's intensity at pixel *p_j,k_* at time instant *k* + 1. Each weight *W_j,k_*_+1_, *j* = 1, 2,…,*N*, is a measure of the similarity between a pair of corresponding points *p_j,k_* and *p_j,k_*_+1_ computed as the convolution of pixel neighborhoods surrounding *p_j,k_* and *P_j,k_*_+1_. The weights *W_j,k_*_+1_, *j* = 1, 2,…, *N*, provide a measure of the quality and reliability of the match and are particularly useful to discard points with lower weights, which are more likely to occur near the boundaries of the SPG when the head turns, as some points will become occluded. Bad tracking of some of the salient points, as depicted in Figure 6, may also occur when the points are occluded by an external object, like the driver's hand, or when weak saliency, due to low textureness or contrast, makes correspondences ambiguous (non-unique).

#### 2.2.4. Tracking Reset Rules

Using the optical flow information *ϕ_j,k_*_+1_, *p_j,k_*, *p_j,k_* and *W_j,k_*, corresponding to the sets *S_k_* and *S_k_*_+1_ of SPG points, the perspective and back-projection mappings П and Γ, and the pose matrices *P_k_* and *P_k_*_+1_, it is possible to formulate the pose estimation problem considering that:

(11) $$\begin{array}{cc} X_{j,k+1}^{W}=M_{k+1}X_{j,k}^{W}, & j=1,2,\ldots ,N \end{array}$$

relates all SPG points $X_{j,k}^{W}\in S_{k}$ to SPG points $X_{j,k+1}^{W}\in S_{k+1}$ through the driver's motion *M_k_*_+1_ at instant *k* + 1. By Equation (3), the SPG points *j* = 1, 2,…, *N* in world coordinates of frame *S^W^* are related to the SPG points in the coordinates of the head frame *S^H^* by:

(12) $$\begin{array}{ccc} X_{j,k}^{W} & = & P_{k}X_{j}^{H}\\ X_{j,k+1}^{W} & = & P_{k+1}X_{j}^{H} \end{array}$$

It is to be noted that the SPG points $X_{j}^{H}$ have been written without a dependency on the time instant, because the SPG is assumed to be a rigid structure attached to the driver's head and, thus, move consistently with *S^H^*. Although this assumption is violated for a limited number of points close to the mouth and eyebrows that move relative to *S^H^* when the driver talks or makes gestures, the distance these points travel and their speed is negligible for practical purposes compared to that of *S^H^* relative to *S^W^* when the driver changes pose. Hence, replacing Equations (12) into (11) yields:

(13) $$P_{k+1}X_{j}^{H}=M_{k+1}P_{k}X_{j}^{H}\Rightarrow P_{k+1}=M_{k+1}P_{k}\Rightarrow M_{k+1}=P_{k+1}P_{k}^{-1}$$

On the other hand, using Equations (5) and (10), the SPG points in camera coordinates *S^C^* are related to the SPG points in world coordinates according to:

(14) $$\begin{array}{ccc} X_{j,k}^{W} & = & CX_{j,k}^{C}=C\Gamma p_{j,k}\\ X_{j,k+1}^{W} & = & CX_{j,k+1}^{C}=C\Gamma p_{j,k+1} \end{array}$$

Equations (13) and (14) allow one to rewrite Equation (11) in terms of the optical flow pair *p_j;k_*, *p_j,k_*_+1_, and the pose matrix *P_k_*_+1_ as:

(15) $$C\Gamma p_{j,k+1}=p_{k+1}p_{k}^{-1}C\Gamma p_{j,k},j=1,2,\ldots ,N$$

and therefore, by Equations (10) and (7):

(16) $$p_{j,k+1}=\Pi C^{-1}P_{k+1}P_{k}^{-1}C\Gamma p_{j,k},j=1,2,\ldots ,N$$

Defining the matrix:

(17) $$T_{k+1}\left(\alpha_{1},\beta_{1},\gamma_{1},\alpha_{2},\beta_{2}\right)\overset{def}{=}\prod C^{-1}P_{k+1}P_{k}^{-1}C\Gamma$$

as the transformation matrix that maps points *p_j;k_* onto *p_j_*_,k+1_ due to a pose change from *P*_k_ to *P_k_*_+1_ dependent on the new pose angles α_1_, β_1_, γ_1_, α_2_ and β_2_, at instant *k* + 1, the pose estimation problem can be formulated as the following optimization problem:

(18) $$\Theta_{k+1}^{*}=\arg\underset{\alpha_{1},\beta_{1},\gamma_{1},\alpha_{2},\beta_{2}}{\min}\sum_{i}w_{j,k+1}\left\|T\left(\alpha_{1},\beta_{1},\gamma_{1},\alpha_{2},\beta_{2}\right)p_{j,k}-p_{j,k+1}\right\|$$

of finding the set of pose angles $\Theta_{k+1}^{*}=(\alpha_{1}^{*},\beta_{1}^{*},\gamma_{1}^{*},\alpha_{2}^{*},\beta_{2}^{*})$ at instant *k* +1 that minimizes the matching error between the pair of points *p_j_*_,k_, *P_j,k_*_+1_ delivered by the LK optical flow computation method considering the knowledge of the pose *P_k_* obtained at time k in the previous iteration. It is to be noted that the transformation matrix *T_k_*_+_*_1_*(*α*_1_, *β*_1_, *γ*_1_, *α*_2_, *β*_2_) in Equation (17) is a 3 × 3 identity matrix when the pose of the driver remains constant, *i.e.*, *P_k_* = *P_k_*_+1_, and the salient points in the image remain static, *i.e.*, *P_j,k_* = *P_j,k_*_+_*_1_, j* = 1, 2,…, N. This means that solving Equation (18) seeks to find the angles $\Theta_{k+1}^{*}$ for *P*_k+1_ that match precisely those of *P_k_*, so that $P_{k+1}P_{k}^{-1}=I_{3\times 3}$, ensuring that the value of the cost function is driven to zero.

The minimization problem Equation (18) is a nonlinear least squares problem, which can be solved by different gradient methods, Newton's method or direct search methods; see, for example, [60,61]. In our implementation, problem Equation (18) was solved using the Levenberg-Marquardt variant of the Gauss-Newton algorithm, as well as the direct search approach by Nelder-Mead. While the Nelder-Mead algorithm may converge to a non-stationary point, in practice, it converged faster than the Levenberg-Marquardt approach and was preferred for this reason. Despite that the theoretical convergence properties of the direct search approaches are often not satisfactory, algorithms, such as Nelder-Mead's, are known to work reasonably well for problems of relatively small dimension (up to 10) [60]. On the other hand, in the case of our problem, the angles are bounded and the initial driver position is known to be constrained to a specific range. This allows one to initialize the algorithm correctly without any danger of converging to a local minima. Moreover, tracking the angles with the extended Kalman filter (EKF) allows one to initialize the solution of Equation (18) at each iteration with the predicted values for angles. This ensures that the search for the solution starts at a close value with respect to the true pose that is being sought. It is also to be noted that the proposed approach considers reset conditions that allow one to restart the process of finding the driver's pose, so the actual risk of a permanently diverging solution is inexistent.

Considering that the pose estimation approach is incremental (*i.e*., the proposed approach estimates a new pose starting from the pose estimated in the previous iteration, as explained in the preceding sections), small errors in the pose estimation occurring in some frames can accumulate over time. To prevent errors from accumulating, two reset conditions are implemented: (i) the system checks if the motion of the driver has not produced angles *α*_1_, *β*_1_, γ_1_, *α_2_* or *β_2_* exceeding ±20° and (ii) the eyes are detected correctly using the Viola-Jones approach at least once every certain number of frames (our implementation checks for a correct eyes detection every 100 frames at 15 fps; the tracking of the eyes on the remaining frames relies on the salient points of the SPG, as explained in the next section). If any of the two conditions is not satisfied, then the tracking system and the pose vector is reset every *n_8_* frames, until both conditions are satisfied again, in which case the tracking system and the pose vector are reset every *n_l_* frames. In our implementation, *n_8_* was set to 100 frames, while *n_l_* was set to 5,000 frames, thus ensuring good tracking results by keeping the cumulative error to a minimum.

### 2.3. Eyes Location and Tracking

The eyes' location is initially obtained using the Viola-Jones approach in a sub-window within the SPG. The Viola-Jones detection approach is not used again during the normal operation of the system, unless certain reset conditions occur (see Section 2.2.4). Once the initial location of the eyes is found, the Viola-Jones approach is not employed on each iteration for two reasons. First, the eye recognition becomes difficult or impossible under partial eye occlusions when the driver stares away from the camera, changes pose or temporarily moves his hands or an object in the line of sight of the camera. On the other hand, the Viola-Jones recognition approach is computationally more expensive than the tracking of the SPG points. Moreover, the SPG provides a set of reference points that allows one to locate the eyes relative to the SPG. Thus, if a few points of the SPG are lost due to occlusions or pose changes, the eyes can still be located relative to the remaining points in the SPG.

Consider the points $X_{j,k}^{W},j=1,2,\ldots ,N$, in the SPG set *S_k_* at instant *k* expressed in the coordinates of *S^W^*, and denote by $e_{k}^{i}\in {\mathbb{R}}^{3},i=l,r$, the location of the left and right eye's pupil in homogeneous image coordinates. By Equation (10), the projection of $e_{k}^{i}\in {\mathbb{R}}^{3}$ onto the SPG, denoted by $E_{k}^{C,i}\in {\mathbb{R}}^{4}$ is given by $E_{k}^{C,i}=\Gamma e_{k}^{i},i=l,r$. The location of the left and right eye relative to the SPG points can then be computed at every instant *k* as:

$$V_{j,k}^{C,i}=E_{k}^{C,i}-X_{j,k}^{C},i=l,r$$

Therefore, when the SPG points are obtained in iteration *k* + 1, the new location of the eyes can be estimated at *k* + 1 from the weighted average of relative displacement of the eyes relative to the new SPG points:

$$\begin{array}{ll} E_{k+1}^{C,i} & =\sum_{j}w_{j,k+1}\cdot \left(X_{j,k+1}^{C}+V_{j,k}^{C,i}\right)\\ & =\sum_{j}w_{j,k+1}\cdot \left(\Gamma p_{j,k+1}+V_{j,k}^{C,i}\right)\\ & =\sum_{j}w_{j,k+1}\cdot \left(\Gamma T_{k+1}p_{j,k}+V_{j,k}^{C,i}\right),i=l,r \end{array}$$

where *T_k_*_+1_ is the transformation matrix defined in Equation (17) representing the motion of the driver at instant *k* + 1 according to the kinematic model Equation (3) and *W_j_*_,k+i_ are the weights of the salient points computed by the LK method at time *k* + 1. Since $\prod E_{k}^{C,i}=e_{k}^{i},\forall k$ pre-multiplying by the perspective projection matrix II, the last equation can be rewritten as:

(19) $$\begin{array}{ll} e_{k+1}^{i} & =\sum_{j}w_{j,k+1}\cdot \left(\prod \Gamma T_{k+1}p_{j,k}+\prod V_{j,k}^{C,i}\right)\\ & =\sum_{j}w_{j,k+1}\cdot \left(\prod \Gamma T_{k+1}p_{j,k}+e_{k}^{i}-p_{j,k}\right)\\ & =\sum_{j}w_{j,k+1}\cdot \left(e_{k}^{i}+\left(T_{k+1}p_{j,k}-p_{j,k}\right)\right),i=l,r \end{array}$$

since ΠΓ = *I*_3×3_. It is to be noted that by Equations (16) and (17), *T_k_*_+1_*p_j,k_* is the expected position of the *j*-th point of the SPG at time *k* + 1, *i.e.*, *p_j_*, *_k_*_+1_ = *T_k_*_+1_*p_j,k_*, and therefore, *T_k_*_+1_*p_j_*_k_ – *p_j,k_* = *p_j,k_*_+1_ – *p_j,k_* = ϕ*_j,k_*_+1_ is the optical flow for the *j*-th SPG point. This result is important, because it implies that the eyes' location within the image can be updated and tracked using the weighted average of the optical flow ϕ*_j,k_* of salient points instead of carrying out a more complex eyes recognition process. This is also advantageous, because in addition to penalizing those points that have lower weights *w_j,k_*_+1_, it means that instead of implementing a Kalman filter to track all the points in the SPG in 3D space, it should suffice to correctly estimate the pose values, *α*_1_, *β*_1_, *γ*_1_, *α*_2_, *β*_2_. In our approach, an EKF is implemented using the kinematic model as a driving process to predict the pose angles together with the new measurements of the eyes' location given by Equation (19) to update the state estimate. It will be shown in the results section that by doing so, the eyes' can be tracked accurately with a high success rate, despite the driver's motion.

### 2.4. Driver Vigilance Measurement and Blink Detection

A driver's state of alert is a combination of factors that include fatigue, drowsiness and distraction from the driving task, while talking to other passengers or persons on a mobile phone. One of the indicators of distraction is the driver's pose, especially whenever the driver's head is not staring forward. While this information can be obtained with the proposed pose estimation and tracking approach, a more critical risk factor is fatigue and drowsiness, since it impairs the driver's attention and diminishes his or her ability to recover from wrong maneuvers until the necessary rest is taken. Thus far, the best measure of fatigue and drowsiness is the percentage of eye closure (PERCLOS) over some period of time [14,15,50,51]. More precisely, PERCLOS is calculated as the ratio between the amount of time the eyes are closed (pupils are 80% or more covered by the eyelids) with respect to the total time lapse:

(20) $$\text{PERCLOS}=\frac{t_{c}}{t_{c}+t_{o}}$$

where *t_c_* is the time the eyes are closed and *t_o_* is the time the eyes are open. This measure is typically computed over a running window lasting one minute.

Several studies ([14,26,27,50,51,62]) have demonstrated that the PERCLOS measure has a high correlation with the level of drowsiness. One of the most important studies was carried out by the Federal Highway Administration of the United States [14] and showed that a person's PERCLOS increases directly with the level of fatigue. The test was made keeping ten subjects awake for 42 h and taking tests of PERCLOS and reaction time every two hours. The results show an average correlation between the reaction time and drowsiness of 0.878. Our results obtained from the tests carried out in a simulator are consistent with the previous studies about PERCLOS reported in the literature.

**Figure 7.**
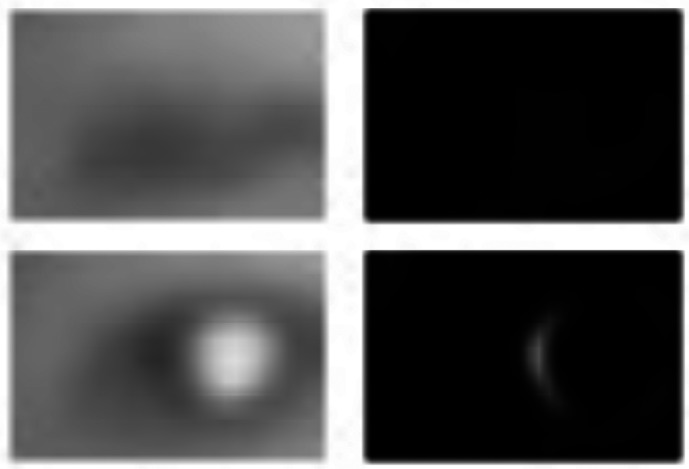
Eye image and filter image for both open and closed eye.

In order to detect blinks and determine whether the eyes are open or closed at every sampling instant *k*, a horizontal Laplacian filter is applied to a neighborhood $N(e_{k}^{i})$ of the image around the eyes central position $e_{k}^{i},i=l,r$. The neighborhood $N(e_{k}^{i}),i=l,r$, has a width and height, respectively, equal to 18% and 33% of the bounding box for the driver's face SPG. The average ${\bar{G}}_{x,k}^{i}$ of the resulting horizontal gradient $G_{x,k}^{i}$ for the image subregion $I_{N(e_{k}^{i})}=\{I(p)|p\in N(e_{k}^{i})\}$, given by:

(21) $${\bar{G}}_{x,k}^{i}=\frac{1}{\left|{ℬ}_{k}^{8}\right|}\sum_{p\in N\left(e_{k}^{i}\right)}G_{x,k}^{i}\left(p\right),i=l,r$$

is calculated to determine the state of the eyes. When the eyes are open, the number of line segments in the vertical direction increases (pupils and corners of the eyes), and therefore, the horizontal gradient $G_{x,k}^{i}(p)$ contains more vertical edges. On the other hand, when the eyes are closed, only the horizontal line of the eyelid is visible, and the response $G_{x,k}^{i}(p)$ to the horizontal gradient applied to $I_{N(e_{k}^{i})}$ is weaker, thus ${\bar{G}}_{x,k}^{i}$ decreases to a minimum when the eye closes. In order to establish whether a blinking has occurred, ${\bar{G}}_{x,k}^{i}$ is compared to a threshold *η* determined experimentally in such a way as to maximize the rate of detection while minimizing the rate of false alarms. Figure 7 shows a closed eye (upper-left), an open eye (lower-left), and the corresponding responses $G_{x,k}^{i}$ to the horizontal Laplacian filter. When the eyes are open, the lower right image clearly shows a more intense response on the vertical segments of the pupil, while the upper right image shows that the intensities in the response are weaker due to the lack of vertical edges.

## 3. Testing Methodology

In order to validate the efficacy of the proposed approach, fifteen volunteers participated in the experiments carried out using a driving simulator. The subjects were divided into three groups for the purpose of comparing PERCLOS measures and reaction times at different levels of fatigue. Five individuals were fully rested (slept the regular 7–8 h), five individuals had minimal rest (slept at least 3.5 h, but not more than 4 h) and five individuals had no rest at all, *i.e.*, were asked not to sleep from one day to the next. The experiments were carried out on Saturdays between 9:00 and 10:00 AM for each participant with sleep deprivation, between 10:00 and 11:00 for participants with partial rest and between 11:00 and 12:00 for participants with full rest, thus requiring a month to collect the data from the fifteen subjects. All subjects were requested to have a regular 7–8 h sleep on the five days previous to the experiment and to record their sleep time from the time they went to bed until the time they woke up. The average sleep time for the group was 7.43 ± 0.61 h. The participants were asked to follow their regular work-day routines, including three meals. All participants declared to have no sleep disorders nor to be under any medication that could produce sleepiness. The fifteen volunteers were all first-time users of the simulator and had only five minutes to practice driving before the initial reaction-time measurement was carried out. The initial reaction time measurements lasted approximately another five minutes and were followed by the actual driving period of forty-five minutes, as explained below.

**Figure 8.**
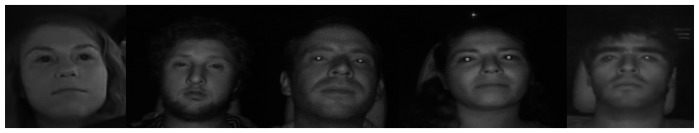
Five of the fifteen participants in the driving experiments under different levels of sleep deprivation.

For the purpose of establishing the influence of drowsiness and fatigue on the tracking and PERCLOS measurements, while minimizing the influence of other factors, like age and phenotype, the participants for this experiment were restricted to a group of similar characteristics consisting of drivers 24–26 years old (six females, nine males). The phenotypes of the group were similar, as shown in Figure 8. Skin colors were in the range from white to brown. Hair color or length was not an issue, since the approach employs a bounding box restricted to the face that encloses the eyes, eyebrows, nose and mouth with a small margin above the eyebrows and below the mouth. In each group, there were three males and two females. One of the three males in each group had a short beard. In each of the three groups, there was one driver that wore prescription polycarbonate glasses and one driver that wore prescription disposable contact lenses. The use of contact lenses had no visible effect on the reflection of the IR illumination. In fact, the transmittance spectrum for most disposable contact lenses is close to 90% for wavelengths above 400 nm, *i.e.*, they block UV light [63] and smaller wavelengths, but are almost transparent to light in the visible and IR spectrum. For uncoated polycarbonate and glass lenses, the transmittance spectrum is similar to that of disposable contact lenses (*cf.* vol. 1, ch. 51D, in [64], or [65]). Therefore, the minor reflections due to the anti-glare coatings were not an impediment to detect the eye blinks. Sunglasses and tinted lenses have lower transmittances for near-infrared, typically 40–60%, depending on the coating and tint. These type of tinted glasses were not considered for our experiments.

The experiments started with a measurement of the participants' reaction time taken before driving. To this end, each subject was required to press a button as fast as possible whenever a green spot would turn red on the simulator's projection screen. This procedure was repeated fifty times using random amounts of time lasting from two to ten seconds between each reaction test.

Once the tests to measure reaction time had been completed, each participant had to drive for forty-five minutes along a rather monotonous track scenario simulating a desert with hills and very few turns. The purpose of the chosen scenario was to induce drivers into falling asleep, while keeping visual distractions to a minimum to prevent arousing the driver's attention.

During the experiments, the driver's reaction time and driving behavior were simultaneously analyzed on-line and recorded with the data capture system implemented to that end. A snapshot of the software implemented to extract salient points and compute the PERCLOS measure is shown in Figure 5. Some of the participants in the driver's fatigue measurement experiment are shown in Figure 8.

The car simulator was built inside a closed lab with no external light sources using a Ford Escape 2009 seat and a Momo Racing Force Feedback Steering Wheel by Logitech, which included gas and brake pedals. A Viewsonic high resolution digital projector was used to project the scenes on a cylindrical projection plane, whose purpose was to immerse the driver into the virtual driving scenario and contribute to the realism perceived by the driver, due to the effects of video motion on the peripheral vision. In other words, the curved backdrop surrounding the driver enhances the persons velocity sensation that would otherwise be very poor if a planar surface would have been used instead. The software employed to create the driving environment is the open source driving simulator Racer [66], which was configured to limit the driving speed to 100 km/h. A sound system was employed to generate the characteristic sound of a regular combustion motor vehicle.

**Figure 9.**
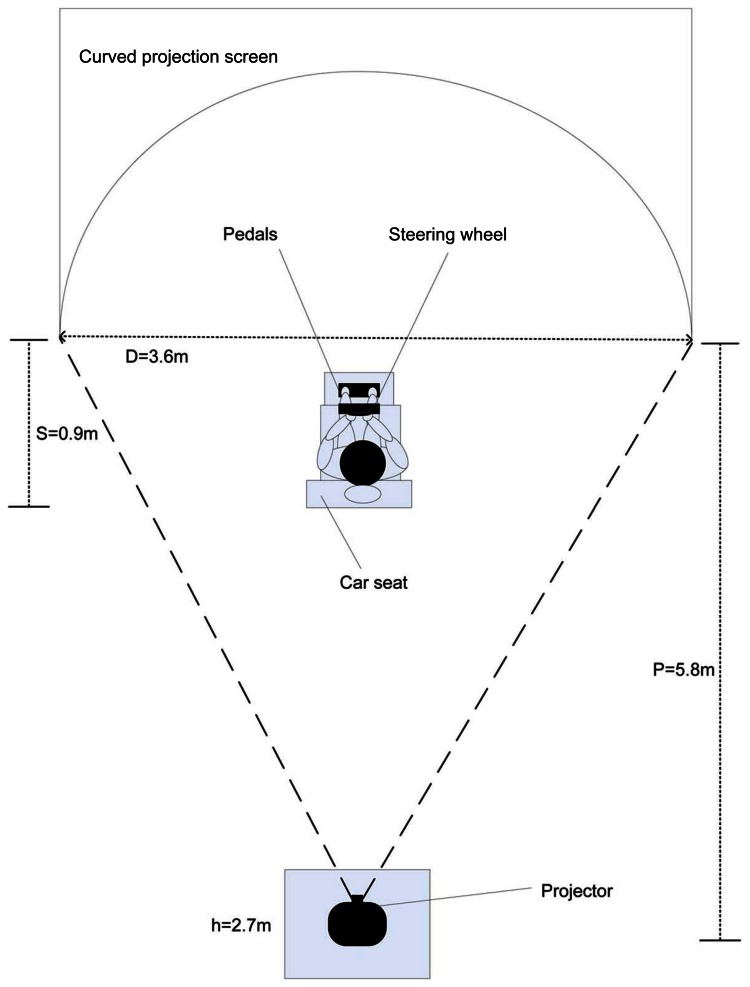
Driving simulator layout.

**Figure 10.**
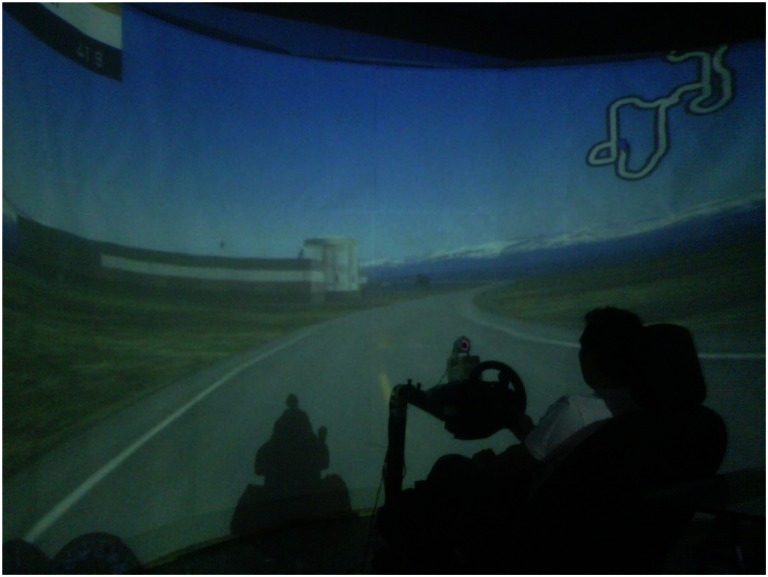
Driving simulator during one of the experiments.

**Figure 11.**
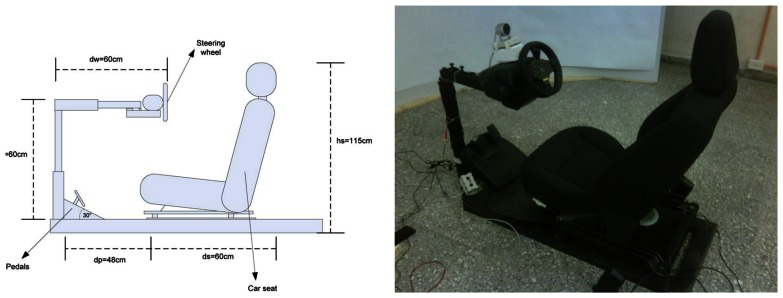
Dimensions and view of the driving seat and steering wheel structure.

The layout of the simulator is illustrated in Figure 9, which shows the semicircular projection screen of 1.8 m radius, the projector located 5.8 m from the projection screen and 2.7 m above the ground, to avoid the car seat structure from casting shadows on the screen. The rear part of the seat structure is 0.9 m away from the center of the semicircular projection screen. This location ensures that the driver field of view subtends the whole projection screen and not just the central portion and also ensures that the driver perceives the virtual world with a scale equivalent to that perceived from a real vehicle, as shown in Figure 10 for one of the driving experiments. From the seating position of the driver in Figure 10, the pavement below the seat and the shadow cast on the screen are not visible. This was possible locating the projector above the screen level and adjusting the keystone effect. The driving seat and its dimensions are shown in Figure 11.

## 4. Results

Eye tracking rates obtained with the proposed approach and two other comparison methods are summarized in Table 1. One of the comparison approaches presented in Table 1 is based on the direct detection of the eyes using the Viola-Jones recognition approach. The second comparison method is based on a salient points tracking approach, but without considering the driver kinematics nor its pose information. This approach had been proposed by the authors in [44] and significantly improved in this work by including the driver kinematics and the proposed scheme for tracking the SPG points, as shown by the results in the last column of Table 1. The results in Table 1 show that a relatively low (38.03% ± 13.57%) tracking rate of the eyes is achieved by direct application of the Viola-Jones technique trained to detect eyes. Compared to the approach based on the direct eyes identification on every frame, the SPG tracking approach significantly improves the success of the eye tracking system with an average success rate of 97.10% ± 2.39. Using the proposed method with the driver's kinematic model, an additional improvement in the tracking rate of 2.31% is possible, yielding on average a failure rate below 1%. The high tracking rate of the proposed approach ensures that the state of the alert measurement system would be able to compute the PERCLOS on practically every image frame, unlike the other approaches that are more sensitive to pose changes and rapid driver movements.

In addition to correctly tracking the driver's motion, an effective drowsiness warning system must be able to differentiate the driver's state of alert. To verify this requirement, the PERCLOS was computed for the different groups of awake, semi-awake and drowsy subjects using the proposed approach. The mean PERCLOS values computed for each group of drivers using windows of 60 seconds are summarized in Table 2 and clearly exhibit an increase for the groups with less hours of sleep. It is also possible to notice that the reaction time increases for the group of drowsy subjects. However, the average reaction time of the fully awake and the semi-drowsy subjects shows little change in contrast to the PERCLOS, which on average is more than doubled. On the other hand, it was observed that the reaction time does not directly correlate with the level of sleep, since some well-rested drivers had average reaction times larger than that of drivers in the drowsy group.

**Figure 12.**
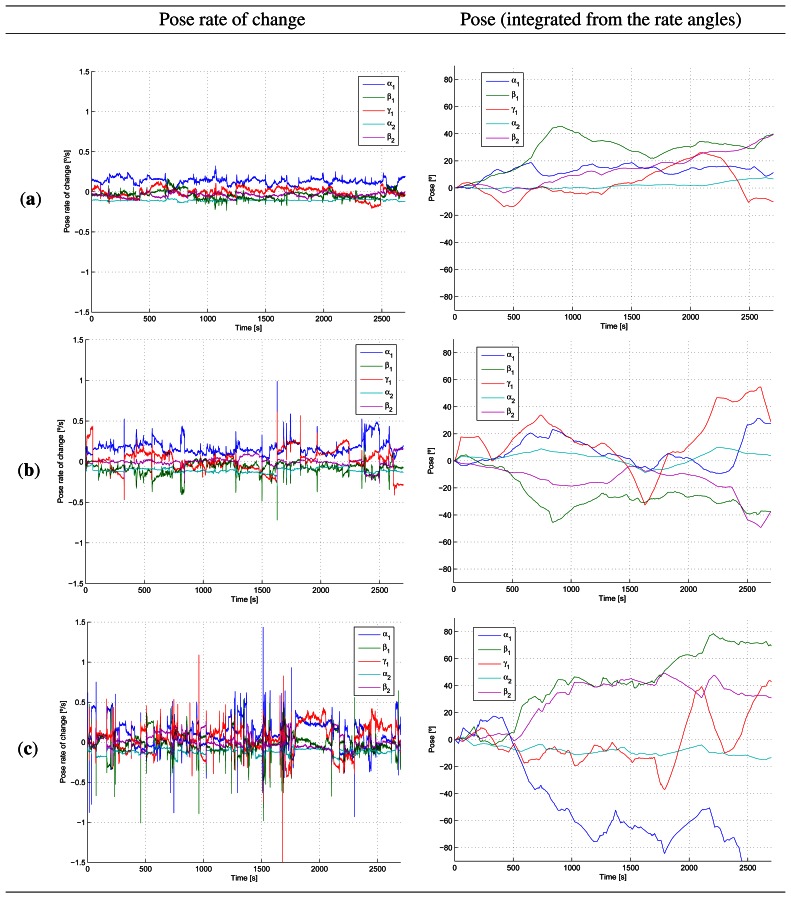
Pose rate of change and pose computed from the integral of the pose rate without reset conditions for the awake (**a**); semi-drowsy (**b**) and drowsy (**c**) drivers, corresponding to test subjects 1, 6 and 11, respectively.

Another indicator of drowsiness is the change in the driver's pose. Table 2 presents the average root mean square (RMS) value of the *pose magnitude* and the *pose rate magnitude* for the different group of drivers. The pose magnitude is computed as the Euclidean norm of the vector of pose angles (*α*_1_, *β*_1_, *γ*_1_, *α*_2_, *β*_2_). Similarly, the pose rate magnitude is calculated as the Euclidean norm of the vector containing the time derivatives of the pose angles. The pose magnitude RMS value for the awake and drowsy drivers was similar, and on average, larger than that of semi-drowsy drivers. However, there is a positive correlation between the mean PERCLOS measure and the RMS value of the rate of change in pose. This is consistent with the knowledge that a drowsy driver will attempt to make fast sudden corrections to deviations from the lane, and it is expectable that the driver will also try to regain the sitting pose quickly, while avoiding to fall asleep. It has been argued, see for example [6,12,13], that monitoring corrections in driving maneuvers and pose changes may not provide information sufficiently in advance to warn the driver. In fact, the evolution of the pose of the driver in time does not seem to provide an indication of fatigue as clearly as the rate of change of the pose, according to our results in Figure 12 for a selection of one awake, one semi-drowsy and one drowsy driver. Due to space limitations, it is not possible to include the plots for the fifteen subjects; however, the curves have similar evolutions for drivers within the same group (awake, semi-drowsy or drowsy). The first column of Figure 12 presents the rate of change of the pose angles, *α*_1_, *β*_1_, *γ*_1_, *α*_2_ and *β*_2_ *versus* time for the awake subject 1 (first row), for the semi-drowsy driver 6 (second row) and the drowsy driver (last row). Clearly, the awake driver presents less sudden rapid motions than the semi-drowsy or the drowsy driver. Integrating the pose rate angles yields the curves in the right column of Figure 12. This second column corresponds to the pose without considering the reset conditions, and therefore, accumulates the measurement errors in the 45 minutes (2,700 seconds) of the experiment. The integrated pose curves for the drowsy driver deviate more from the starting pose than those of the semi-drowsy or fully awake drivers, due to the larger number of sudden pose corrections. We observed that awake drivers tended to seek a more comfortable sitting position after a while of driving or simply changed position because of boredom. However, awake drivers kept their position for longer periods. On the other hand, drowsy driver's were struggling not to fall asleep, seemed also more concerned about not failing the test and, therefore, would move quickly to regain control of themselves.

**Figure 13.**
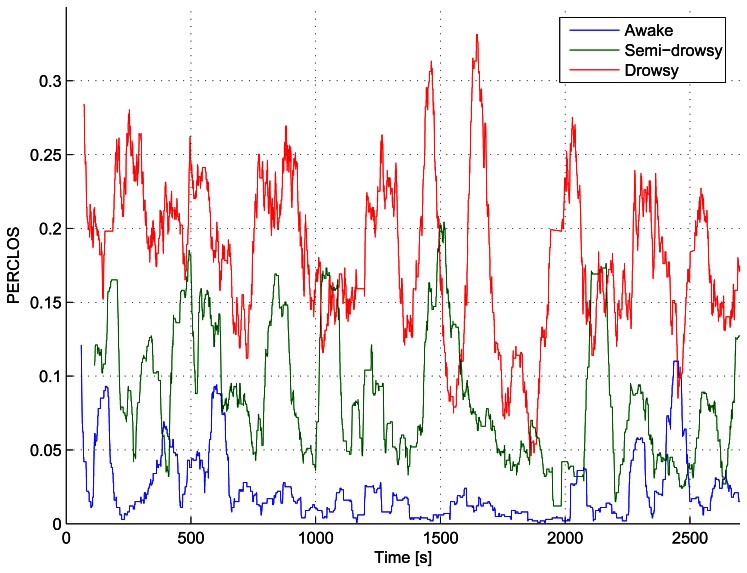
PERCLOS for the awake (**a**); semi-drowsy (**b**) and drowsy (**c**) drivers, corresponding to test subjects 1, 6 and 11, respectively.

**Figure 14.**
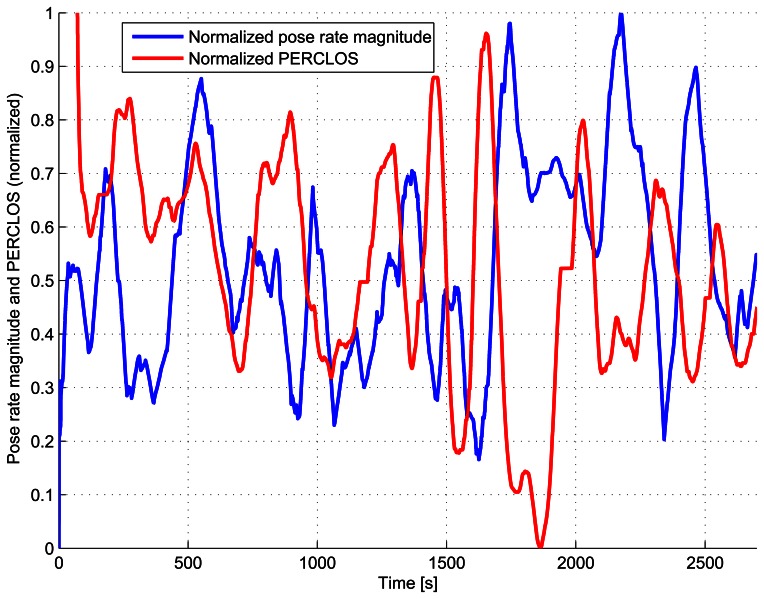
Normalized pose rate magnitude and normalized PERCLOS measure for drowsy subject 11, both smoothed with a moving average filter using a window of 80 seconds.

The PERCLOS curves for drivers 1, 6 and 11 are shown in Figure 13. Apart from the fact that the PERCLOS curve for the drowsy driver has an appreciably larger average value than that of the semi-drowsy and awake drivers, the PERCLOS of the drowsy driver increases precisely before instants in which the drowsy driver makes sudden motions (e.g., seconds 900, 1,500, 1,700, 2,000, 2,400). This can also be seen in Figure 14, which shows the evolution of the normalized magnitude (Euclidean norm) of the pose rate vector and the normalized PERCLOS measure for the drowsy driver 11. Both, the normalized magnitude of the pose rate vector and the normalized PERCLOS measure, have been smoothed using a moving average filter with a window spanning 80 seconds and normalized to values in the range [0,1] to facilitate the comparison. While there does not seem to be any straight forward connection between the amplitude of the peaks in the normalized PERCLOS and the amplitude of those in of the normalized pose rate magnitude, from Figure 14, it is possible to observe that the peaks in the normalized PERCLOS precede the majority of peaks of the normalized pose rate magnitude. This fact that was also observed for the other semi-drowsy and drowsy drivers strengthens the support for PERCLOS as a measure that has more predictive value than other physiological cues that can be measured in a non-invasive manner, such as the driver's pose variations or steering behavior.

The previous results, together with the fact that the 95% confidence interval for the average PERCLOS value is very narrow (see Table 2), confirm that the PERCLOS measure is more reliable for correctly discriminating the different fatigue levels. This conclusion is also supported by the PERCLOS normal distribution curves plotted in Figure 1 for each group of drivers using the computed PERCLOS mean and standard deviation values. Figure 1 shows a clear difference in the mean PERCLOS for the different levels of drowsiness with non-overlapping confidence intervals. The normal distribution curves for each group of drivers can be assumed to specify the probability distributions for each class and used to select the class for which the measurement has the highest probability of belonging to. The normal distribution curves were used to obtain the threshold values presented in Table 3, which are needed to classify the driver's level of alert.

## 5. Conclusions

A non-invasive sensing approach for driver fatigue and attention measurement was presented. The novelty of the approach is in the use of a kinematic model of the driver's motion and a grid of salient points tracked using the Lukas-Kanade optical flow method. The advantage of this approach is that it does not require one to directly detect the eyes, and therefore, if the eyes are occluded or not visible from the camera when the head turns, the system does not loose the tracking of the eyes or the face, because it relies on the grid of salient points and the knowledge of the driver's motion model, which is useful for computing and predicting the pose of the driver. Another contribution of this approach is that it employs the observed interpupillary distance to estimate (up to a scale factor) how far the driver is from the camera. In other words, the approach does not require a stereoscopic system to resolve the relative motion of the driver. Moreover, the kinematic motion model for a driver with five degrees of freedom allows one to implement an extended Kalman filter that simplifies the tracking of the points in the image space. The results show that the tracking rate improves from 38.03 ± 13.57% to 97.10 ± 2.39%, when the salient points are used instead of attempting to perform the eyes recognition using the Viola-Jones approach. An additional improvement from 97.10 ± 2.39% to 99.41 ± 1.31% is possible using the kinematic model with the extended Kalman filter.

The experiments performed involved a group of 15 subjects, five of which were asked to stay awake for more than 24 hours before the driving tests in the simulator, another five were asked to sleep only four hours the night before the test and five were asked to have a full eight hours rest. The computation of the percentage of time the eyes are closed covering at least 80% or more of the pupil (PERCLOS) for the different group of subjects delivers a measure that is consistent with the drivers' level of drowsiness. The results show that these three groups have a PERCLOS with a sufficiently small variance for classification purposes, *i.e.*, the PERCLOS measure can be used to effectively distinguish and detect the level of fatigue associated to the lack of rest. It was found that the group of subjects in the awake state presents a mean PERCLOS value of 0.0320 ± 0.0021 (C.I. 95%) with a standard deviation of 0.0074, while subjects in the drowsy state have a mean PERCLOS of 0.1799 ± 0.0146 (C.I. 95%) with a standard deviation of 0.0499, thus exhibiting a difference between the two states significant enough that can be used by the drowsiness and attention system to warn the driver about having reached dangerous fatigue levels, which could lead to an imminent accident, unless proper rest is taken.

In summary, the results demonstrate that the proposed system provides a solution for drowsiness and attention sensing that is reliable and more robust to occlusions or driver pose changes that often affect approaches based on the direct tracking of the eyes. In addition to the measurement of fatigue and drowsiness, the pose tracking capability of the proposed approach has potential applications in distraction assessment and alerting of machine operators, particularly of large construction and mining machinery, which is a subject of the authors' ongoing research. This study considered a group of similar participants in age and daily routine. The analysis of fatigue variation across gender, age or phenotype was not in the scope of the current work. These aspects, together with a detailed study of optimal IR illumination for people wearing tinted glasses, are also part of the authors' ongoing long-term research efforts.

## Figures and Tables

**Table 1.** Eye tracking results with the proposed approach.

**Subject**	**Frames**	**Tracking Rate [%]**		

		**Viola-Jones Eyes Recognition**	**SPG Tracking without Driver Kinematics ([44])**	**SPG Tracking with Driver Kinematics**
1	2768	22.24	93.39	100.00
2	6122	43.16	99.46	100.00
3	5219	21.18	98.58	100.00
4	3310	58.29	94.46	96.79
5	5253	44.33	98.50	99.48

Mean ± 95% C.I.		38.03 ± 13.57	97.10 ± 2.39	99.41 ± 1.31

**Table 2.** PERCLOS average, standard deviation, 95% confidence interval for the different subjects (1–5 awake, 6–10 semi-drowsy and 11–15 drowsy) and reaction time, pose and pose rate-of-change for the ensemble of awake, semi-drowsy and drowsy subjects.

Subject A/S/D*	**PERCLOS**		

	**Awake**	**Semi-Drowsy**	**Drowsy**
1/6/11	0.0281	0.1089	0.1891
2/7/12	0.0329	0.0660	0.1768
3/8/13	0.0291	0.0905	0.2099
4/9/14	0.0444	0.1088	0.0975
5/10/15	0.0255	0.0663	0.2262

Mean	0.0320	0.0881	0.1799
Std. Dev.	0.0074	0.0214	0.0499
95% C.I.	±0.0021	±0.0084	±0.0146

Avg. Reaction Time [ms]	199.0	204.6	262.5
Avg. Pose Magnitude RMS [°]	61.7	51.1	67.7
Avg. Pose Rate RMS [°/*s*]	0.13	0.20	0.25

* Subject A = awake; S = semi-drowsy; D = drowsy.

**Table 3.** PERCLOS threshold values for driver state classification using 60 seconds windows.

**Driver state**	**Minimum PERCLOS**	**Maximum PERCLOS**
Fully awake	0.000	0.048
Semi-drowsy	0.048	0.125
Drowsy-driver	0.125	1.000
